# Identifying predictor factors for asthma using machine learning: evidence from the English Longitudinal Study of Ageing

**DOI:** 10.1186/s12889-026-28253-9

**Published:** 2026-07-03

**Authors:** Zhao Chen Yang, Zhou Xiao

**Affiliations:** Department of Respiratory/Gerontology, Xi’an Honghui Hospital, Honghui Hospital, Xi’an Jiaotong University, Xi’an, China

**Keywords:** Asthma, Predictor factors, Machine learning, SHapley additive exPlanations, Predictive model

## Abstract

**Background:**

Asthma is a common chronic inflammatory airway disease. Accumulating evidence highlights the roles of demographic, lifestyle, and comorbidity factors in the risk of asthma. This study aimed to identify predictor factors of asthma using machine learning approaches.

**Methods:**

Data were obtained from the 10th wave (2021–2023) of the English Longitudinal Study of Ageing (ELSA). Participants aged ≥ 50 years with complete information on asthma status and relevant variables were included. Baseline characteristics were compared between asthma and control groups. Subsequently, Least Absolute Shrinkage and Selection Operator (LASSO) regression was used to identify candidate variables. Eight machine learning algorithms were developed and compared to evaluate diagnostic performance. The optimal model was selected and used to determine key variables. Additionally, SHapley Additive exPlanations (SHAP) analysis was applied to interpret variable contributions. Finally, a nomogram was constructed based on the key variables.

**Results:**

A total of 3429 participants (535 asthma cases) were analyzed. Asthma was significantly associated with 19 baseline variables. LASSO regression retained 14 candidate variables. Among eight machine learning models, the Bagging Tree (BT) model achieved the highest diagnostic performance (micro-averaged area under the curve (AUC) = 0.856; macro-averaged AUC = 0.881). SHAP analysis identified alcohol consumption, marital status, and disease lung as the most influential variables. A total of 11 key variables were identified by the BT model, including marital status, vigorous physical activity, moderate physical activity, alcohol consumption, frequency of feeling isolated, depression, headache, activity limitations, disease lung, arthritis, and psychiatric disease. The nomogram showed good calibration (Hosmer-Lemeshow test *p* = 0.0857), but its discriminatory ability was moderate (AUC = 0.662).

**Conclusions:**

This study demonstrated that socio-behavioral factors, psychological distress, and respiratory comorbidities played important roles in asthma risk stratification. Machine learning with multidimensional variables offers a useful exploratory framework for identifying potential predictor factors and generating hypotheses for asthma prevention, although its predictive accuracy remains moderate.

**Supplementary Information:**

The online version contains supplementary material available at https://doi.org/10.1186/s12889-026-28253-9.

## Introduction

Asthma is a common chronic inflammatory airway disease characterized by recurrent episodes of wheezing, breathlessness, chest tightness, and coughing, with significant impacts on patients’ quality of life and a substantial socioeconomic burden [1–3]. Globally, the prevalence of asthma continues to rise, influenced by a complex interplay of genetic predispositions, environmental exposures, and lifestyle factors [4–8]. Previous research indicates that demographic characteristics (e.g., sex), lifestyle habits (e.g., physical activity), and various health-related comorbidities (e.g., obesity, respiratory and pulmonary conditions) are closely associated with asthma development [9–14].Currently, the clinical management of asthma primarily centers on anti-inflammatory controller medications such as inhaled corticosteroids, aiming to alleviate symptoms and prevent acute exacerbations [15]. However, this constitutes symptomatic treatment that cannot reverse the natural course of the disease or achieve a cure; some patients exhibit poor treatment response and develop refractory asthma, and adherence to long-term medication, especially among middle-aged and elderly populations with multiple comorbidities, is often difficult to ensure [16,17]. Consequently, the systematic identification and integration of multidimensional factors are crucial for elucidating the underlying etiological mechanisms of asthma, optimizing risk stratification, and advancing precise prevention and personalized intervention strategies.

The English Longitudinal Study of Ageing (ELSA), funded by the UK Economic and Social Research Council (ESRC), is a nationally representative cohort study designed to track and assess changes in health, social, economic, and psychological domains among the middle-aged and older population [18–20]. Adhering to stringent ethical standards, ELSA has become a vital database for international aging research, providing rich data support for investigating the epidemiological characteristics and risk factors of chronic diseases, including asthma [21–24]. It is precisely the multidimensional nature of ELSA data that renders it particularly suitable for this study. By capturing the holistic context of participants beyond clinical indicators, ELSA enables us to innovatively examine how upstream factors such as social connectedness, health behaviors, and psychological distress collectively influence the risk of chronic diseases like asthma, thereby addressing a key gap in the current predominantly biomedically oriented research landscape.

Leveraging data from the 10th wave (2021–2023) of ELSA, this study systematically analyzed potential factors closely associated with asthma in a cohort of eligible individuals aged 50 years and above. We initially compared baseline clinical characteristics and employed Least Absolute Shrinkage and Selection Operator (LASSO) regression to screen candidate variables significantly associated with asthma. Subsequently, eight distinct machine learning models were constructed and compared to evaluate their predictive performance for asthma diagnosis. The best-performing model was selected to identify key variables strongly linked to asthma. Furthermore, Shapley Additive exPlanations (SHAP) analysis was applied to interpret the optimal model, clarifying the role of each key variable in asthma risk stratification. Additionally, a nomogram model was developed based on these key variables for visual risk prediction and underwent calibration validation. This study not only methodologically highlights the advantages of machine learning in identifying predictor factors and modeling risk for complex chronic diseases like asthma but also provides a scientific reference for early screening and individualized prevention in practical terms, holding significant clinical application value and public health implications.

## Materials and methods

### Data source and processing

The data for this study were obtained from the ELSA database (https://www.elsa-project.ac.uk), a nationally representative longitudinal cohort study funded by the ESRC in the UK. Non-sensitive data from ELSA were accessed through the UK Data Service platform (https://beta.ukdataservice.ac.uk/datacatalogue/series/series?id=200011) following approval of the application. ELSA adheres to stringent ethical standards, with approval from the UK National Research Ethics Service or the Declaration of Helsinki, and all participants provided written informed consent prior to enrollment. The database included comprehensive data collected approximately biennially through detailed face-to-face interviews, self-completed questionnaires, physical measurements, and biomarker assessments. These covered domains such as health status, functional ability, cognitive function, mental health, social engagement, family structure, wealth accumulation, and retirement planning. For this analysis, data from the 10th wave (2021–2023) of ELSA were utilized (accessed on 15 July 2025). Participants aged less than 50 years (the minimum enrollment age for ELSA, *n* = 221), as well as those with missing asthma information (*n* = 5) or incomplete/refused responses for any potential predictive variable (*n* = 3934), were excluded. For individuals with missing data as described above, we simply excluded them from the final analysis. After applying these exclusion criteria, a final cohort of 3,429 participants was included in the study.

### Definition of variables

The outcome variable was physician-diagnosed asthma, which was extracted from the ELSA core chronic disease survey item with the original label “Chronic: diagnosed asthma”. Participants were asked to report whether they had ever received a confirmed diagnosis of asthma from a qualified medical physician (general practitioner or specialist in respiratory medicine). The variable was dichotomized as a binary indicator: “yes” (self-reported physician-diagnosed asthma, asthma group) or “no” (no physician-diagnosed asthma, control group). In this study, predictor variables were initially screened based on a systematic review of asthma epidemiology literature, aiming to cover multiple dimensions that may influence asthma risk in middle-aged and older populations. The selection criteria were as follows: Demographic factors (e.g., age, sex, marital status): have been widely reported to be associated with the prevalence and control of asthma [25–27]. Lifestyle and behavioral factors (e.g., smoking, alcohol consumption, physical activity at all levels of intensity): these are intervenable factors that have a direct effect on inflammation levels and airway responsiveness [28–30]. Psychosocial factors (e.g., loneliness, depression, social isolation): Within the framework of psychoneuroimmunology, chronic stress and negative emotions may exacerbate systemic and airway inflammation via activation of the hypothalamic–pituitary–adrenal axis [31–33]. Comorbid conditions (e.g., mental disorders): Asthma often coexists with other chronic inflammatory diseases, suggesting shared pathophysiological pathways [34]. For the final analysis, 2 continuous variables and 42 categorical variables that were available in Wave 10 of the ELSA dataset and met the above dimensions were included (detailed in Additional file 1).

### Baseline characteristics

To investigate the relationship between asthma and potential predictive variables, baseline characteristics were stratified by asthma status (control vs. asthma group) and summarized using the “tableone” package (v 0.13.2) [35]. Categorical variables (e.g., sex, comorbidities) were expressed as percentages and compared using a weighted chi-square test. Continuous variables (age and number of children) were summarized as mean ± standard deviation (SD) or median and interquartile range (IQR) and compared using Student’s t-test. Variables with *p* < 0.05 were considered significantly associated with asthma.

### Machine learning and SHAP interpretability analysis

LASSO regression was employed to identify candidate variables associated with asthma. This method constructs a penalized regression model to shrink regression coefficients toward zero, mitigating multicollinearity and overfitting in multivariate regression models. The analysis was performed using the variables significantly associated with asthma using the “glmnet” package (v 4.1.8) [36]. The response variable was specified as binomial, with the penalty parameter α set to 1. The optimal lambda value was determined through 10-fold cross-validation. Feature selection was carried out independently within each fold of cross-validation.

To systematically evaluate the diagnostic performance of LASSO-selected candidate variables for asthma, eight machine learning algorithms (each machine learning algorithm is based on the candidate variables screened out by LASSO) were implemented using the following R packages: Logistic Regression (LR) was implemented using the “stats” package (v 4.3.1) [37] (family = binomial(link = “logit”), data = data). Decision Tree (DT) was constructed using the “caret” package (v 6.0.94) [38] (data = data, method = “class”). Support Vector Machine (SVM) was trained via the “e1071” package (v 1.7.14) [39] (type = “C-classification”, kernel = “radial”, probability = TRUE). Random Forest (RF) was built with the “randomForest” package (v 4.7.1.1) [40] (ntree = 500, seed = 12345). Bagging Tree (BT, an ensemble learning method based on the Bagging strategy, taking random forests as the foundation, builds multiple independent decision trees through Bootstrap resampling, and finally integrates the prediction results of all decision trees to improve the prediction accuracy and stability of the model) was achieved using the “randomForest” package (v 4.7.1.1, mtry = ncol(data)-1, ntree = 500, seed = 12345). Linear Discriminant Analysis (LDA) was executed via the “MASS” package (v 7.3.60) [41] (prior = c(0.5, 0.5)). Neural Network (NNET) was trained using the “nnet” package (v 7.3.19) [42] (size = 5, trace = FALSE, seed = 12345). Stochastic Gradient Boosting Tree (SGBT) was implemented with the “gbm” package (v 2.1.8) [43] (distribution = “bernoulli”, n.trees = 500, interaction.depth = 3, shrinkage = 0.01, bag.fraction = 0.7). Grid search was adopted to explore the optimal hyperparameter combinations for each algorithm. To ensure model generalizability, a 10-fold cross-validation approach was employed. The dataset was partitioned into 10 equally sized subsets. For each iteration, one subset was retained as the test set, while the remaining nine subsets were used for model training. This process was repeated 10 times, ensuring every subset served as the test set once. To ensure consistent class distribution between the training and test sets, stratified sampling was adopted for cross-validation. Each fold maintained the same ratio of asthma patients to control subjects as in the original dataset, preventing the test set from being dominated by the control group. Performance metrics—including area under the curve (AUC), logarithmic loss, sensitivity, specificity, accuracy, F1 score, Brier score, positive/negative predictive value (PPV/NPV), and average features used—were averaged across all folds. Model performance was visualized via receiver operating characteristic (ROC) curves using the “pROC” package (v 1.18.5) [44]. The model with optimal overall performance across these metrics was selected as the final model, and the candidate variables identified by this best-performing model were defined as key variables. To comprehensively evaluate model performance, this study calculated two AUC metrics: micro-average AUC and macro-average AUC. The micro-average AUC aggregates the prediction results from all cross-validation folds and calculates a single value, reflecting the model’s overall discriminative ability; it is more heavily influenced by the class with the larger sample size; In contrast, macro-average AUC is calculated by first determining the AUC for each fold and then taking their arithmetic mean, which better reflects the model’s stability across different data subsets. These two metrics are often used in conjunction in class-imbalanced datasets to provide complementary information [45, 46].

To interpret individual predictions and validate model decision logic, SHAP values were computed for the optimal machine learning model using the “fastshap” package (v 0.1.1) [47]. This approach quantified the contribution of each variable to asthma risk predictions at the individual participant level and identified directionality (positive/negative association) and magnitude of key variable in individual risk stratification.

### Construction and validation of the asthma risk nomogram

A nomogram for predicting asthma incidence was developed based on key variables identified by the optimal machine learning model. The “rms” package (v 6.5.0) [48] was utilized to construct the nomogram, with asthma status (whether) as the dependent variable and optimal model-selected variables as independent variables. To evaluate the nomogram’s predictive accuracy, the following validation steps were performed. A calibration curve was plotted using the “regplot” package (v 1.1) [49] to evaluate the agreement between predicted and observed asthma probabilities, and its goodness-of-fit was estimated by the Hosmer-Lemeshow (HL) test (*p* > 0.05). In addition, clinical utility was determined through decision curve analysis (DCA) conducted via the “rmda” package (v 1.6) [50] with a net benefit > 0 indicating clinical utility of the nomogram. Discriminatory power was evaluated through receiver operating characteristic (ROC) analysis, performed using the “pROC” package (v 1.18.5), where the AUC was computed to quantify the model’s ability to distinguish between asthma and non-asthma cases. Finally, a confusion matrix was generated, and several key metrics were calculated to comprehensively evaluate the classification performance of the nomogram. These metrics included True Positives (TP), which represented asthma cases correctly classified as positive; True Negatives (TN), which denoted non-asthma controls correctly classified as negative; False Positives (FP), which were non-asthma controls misclassified as asthma; and False Negatives (FN), which were asthma cases misclassified as non-asthma. Derived performance metrics included Recall (Sensitivity), calculated as Recall = TP/(TP + FN); Precision, calculated as Precision = TP/(TP + FP); and the F1 Score, calculated as F1 = 2×(Precision×Recall)/(Precision+Recall). All calculations were performed using the “caret” package (v 6.0.94).

### Statistical analysis

Data extraction and cleaning were performed using the “haven” package (v 2.5.4) [51] and the “tidyr” package (v 1.3.1) [52]. All analyses were performed using R software (v 4.2.2). Statistical significance was defined as *p* < 0.05.

## Results

### Asthma diagnosis was significantly associated with demographic, lifestyle, psychological, and health-related factors

To examine the associations between baseline demographic, lifestyle, and health-related factors and asthma diagnosis, participants were categorized into two groups based on physician-diagnosed asthma status: a control group (*n* = 2894) and an asthma group (*n* = 535). Significant associations were observed for 19 variables (*p* < 0.05, Table 1), indicating robust links to asthma diagnosis. In the asthma group, gender distribution revealed 214 males (40.0%) and 321 females (60.0%). Marital status varied, with 50 participants single (9.3%), 254 married (47.5%), 80 remarried (15.0%), 16 separated (3.0%), 80 divorced (15.0%), and 55 widowed (10.3%). For physical activity, vigorous levels were reported as follows: 71 participants engaged multiple times per week (13.3%), 40 once per week (7.5%), 29 one to three times per month (5.4%), and 395 rarely or never (73.8%). Moderate physical activity showed 285 participants active multiple times per week (53.3%), 67 once per week (12.5%), 33 one to three times per month (6.2%), and 150 rarely or never (28.0%). Alcohol consumption patterns included 91 participants drinking almost daily (17.0%), 25 five to six days per week (4.7%), 72 three to four days per week (13.5%), 109 one to two days per week (20.4%), 49 one to two times per month (9.2%), 38 every few months (7.1%), 54 one to two times per year (10.1%), and 97 never drinking (18.1%). Psychological factors demonstrated notable associations: feelings of being ignored were reported as almost never by 290 participants (54.2%), occasionally by 206 (38.5%), and frequently by 39 (7.3%). Feelings of isolation were almost never experienced by 291 (54.4%), occasionally by 185 (34.6%), and frequently by 59 (11.0%). Additionally, 451 participants reported loneliness (84.3%), and 439 reported depression (82.1%). Health-related comorbidities were prevalent, with 233 reporting headaches (43.6%), 324 exhibiting activity limitations (60.6%)—a composite variable derived from 15 sub-variables (e.g., dressing, bathing, and mobility, where any sub-variable score of 1 resulted in a composite score of 1)—417 diagnosed with disease lung (77.9%), 247 with arthritis (46.2%), 464 with osteoporosis (86.7%), 432 with cancer (80.7%), and 400 with psychiatric disease (74.8%). These results indicated that asthma diagnosis was significantly associated with a range of factors, including gender disparities, marital dynamics, physical inactivity, alcohol use patterns, psychological distress, and comorbid conditions such as respiratory, musculoskeletal, and mental health disorders.

**Table 1:** Clinical characteristics

	level	control group	asthma group	*p*
*n*		2894	535	
age (mean (SD))		68.92 (8.98)	68.43 (9.11)	0.249
sex (%)	Male	1384 (47.8)	214 (40.0)	0.001
	Female	1510 (52.2)	321 (60.0)	
ethnicity (%)	White	90 (3.1)	12 (2.2)	0.344
	Non white	2804 (96.9)	523 (97.8)	
marital status (%)	Single	286 (9.9)	50 (9.3)	0.021
	Married	1546 (53.4)	254 (47.5)	
	Remarried	370 (12.8)	80 (15.0)	
	Separated	44 (1.5)	16 (3.0)	
	Divorced	351 (12.1)	80 (15.0)	
	Widowed	297 (10.3)	55 (10.3)	
vigorous activity (%)	Multiple times every week	643 (22.2)	71 (13.3)	< 0.001
	Once every week	264 (9.1)	40 (7.5)	
	1–3 times every month	228 (7.9)	29 (5.4)	
	Rarely or never	1759 (60.8)	395 (73.8)	
moderate activity (%)	Multiple times every week	1849 (63.9)	285 (53.3)	< 0.001
	Once every week	408 (14.1)	67 (12.5)	
	1–3 times every month	156 (5.4)	33 (6.2)	
	Rarely or never	481 (16.6)	150 (28.0)	
mild activity (%)	Multiple times every week	2204 (76.2)	385 (72.0)	0.051
	Once every week	321 (11.1)	61 (11.4)	
	1–3 times every month	114 (3.9)	22 (4.1)	
	Rarely or never	255 (8.8)	67 (12.5)	
still smoking (%)	Yes	2522 (87.1)	470 (87.9)	0.705
	No	372 (12.9)	65 (12.1)	
drinking (%)	Almost daily	479 (16.6)	91 (17.0)	0.002
	5–6 days every week	212 (7.3)	25 (4.7)	
	3–4 days every week	466 (16.1)	72 (13.5)	
	1–2 days every week	667 (23.0)	109 (20.4)	
	1–2 days every month	286 (9.9)	49 (9.2)	
	Once every few months	199 (6.9)	38 (7.1)	
	Once every 1–2 years	226 (7.8)	54 (10.1)	
	None in the past year	359 (12.4)	97 (18.1)	
number child(mean (SD))		1.82 (1.71)	1.82 (1.86)	0.949
lack companionship (%)	Almost never	1748 (60.4)	301 (56.3)	0.101
	Occasionally	942 (32.6)	185 (34.6)	
	Often	204 (7.0)	49 (9.2)	
neglected frequencies (%)	Almost never	1728 (59.7)	290 (54.2)	0.011
	Occasionally	1028 (35.5)	206 (38.5)	
	Often	138 (4.8)	39 (7.3)	
isolated frequency (%)	Almost never	1823 (63.0)	291 (54.4)	< 0.001
	Occasionally	901 (31.1)	185 (34.6)	
	Often	170 (5.9)	59 (11.0)	
frequency harmony (%)	Almost never	374 (12.9)	70 (13.1)	0.993
	Occasionally	1497 (51.7)	277 (51.8)	
	Often	1023 (35.3)	188 (35.1)	
loneliness (%)	Yes	2579 (89.1)	451 (84.3)	0.002
	No	315 (10.9)	84 (15.7)	
depression (%)	Yes	2571 (88.8)	439 (82.1)	< 0.001
	No	323 (11.2)	96 (17.9)	
headache (%)	Yes	1637 (56.6)	233 (43.6)	< 0.001
	No	1257 (43.4)	302 (56.4)	
activity limitations (%)	Yes	2186 (75.5)	324 (60.6)	< 0.001
	No	708 (24.5)	211 (39.4)	
disease lung (%)	Yes	2716 (93.8)	417 (77.9)	< 0.001
	No	178 (6.2)	118 (22.1)	
disease arthritis (%)	Yes	1662 (57.4)	247 (46.2)	< 0.001
	No	1232 (42.6)	288 (53.8)	
disease osteoporosis (%)	Yes	2627 (90.8)	464 (86.7)	0.005
	No	267 (9.2)	71 (13.3)	
disease cancer (%)	Yes	2451 (84.7)	432 (80.7)	0.026
	No	443 (15.3)	103 (19.3)	
disease parkinson (%)	Yes	2875 (99.3)	534 (99.8)	0.317
	No	19 (0.7)	1 (0.2)	
disease psychiatric (%)	Yes	2471 (85.4)	400 (74.8)	< 0.001
	No	423 (14.6)	135 (25.2)	
disease alzheimer (%)	Yes	2884 (99.7)	533 (99.6)	1
	No	10 (0.3)	2 (0.4)	
heart_failure (%)	Yes	2831 (97.8)	525 (98.1)	0.772
	No	63 (2.2)	10 (1.9)	
heart_murmur (%)	Yes	2740 (94.7)	503 (94.0)	0.606
	No	154 (5.3)	32 (6.0)	
heart_rhythm (%)	Yes	2458 (84.9)	433 (80.9)	0.023
	No	436 (15.1)	102 (19.1)	
diabetes (%)	Yes	2532 (87.5)	441 (82.4)	0.002
	No	362 (12.5)	94 (17.6)	
stroke (%)	Yes	2746 (94.9)	491 (91.8)	0.006
	No	148 (5.1)	44 (8.2)	

### BT modeling and SHAP analysis highlighted marital status, alcohol use, and disease lung as pivotal factors for asthma

**Fig. 1:**
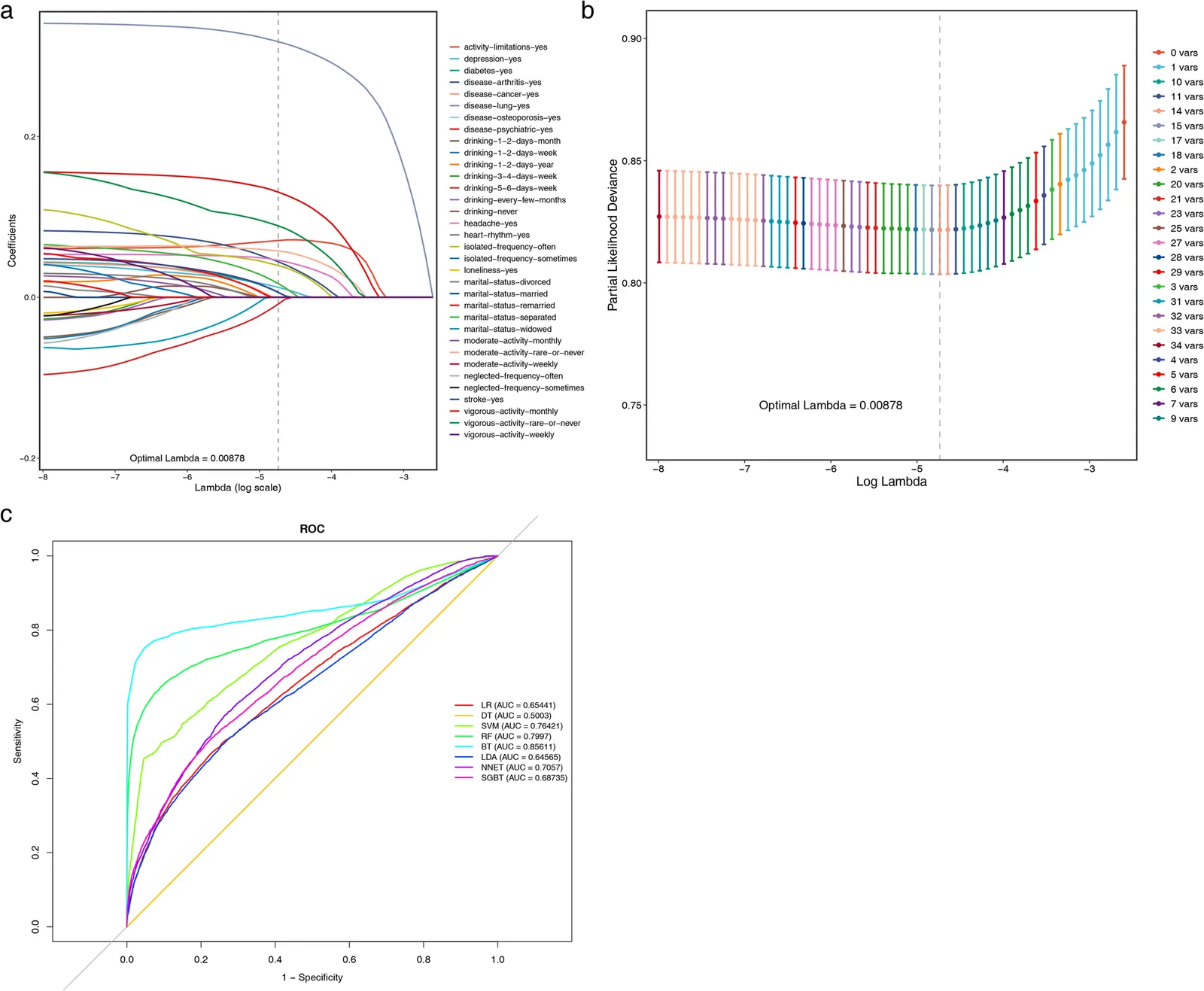
Screening of candidate variables and selection of the optimal model. **a**-**b** Candidate variables were screened using the LASSO regression model. **c** ROC curves of eight machine learning algorithms. The AUC value reported in the figure is the micro-average value, that is, all the folded predictions are combined first, and then an AUC value is calculated

**Fig. 2:**
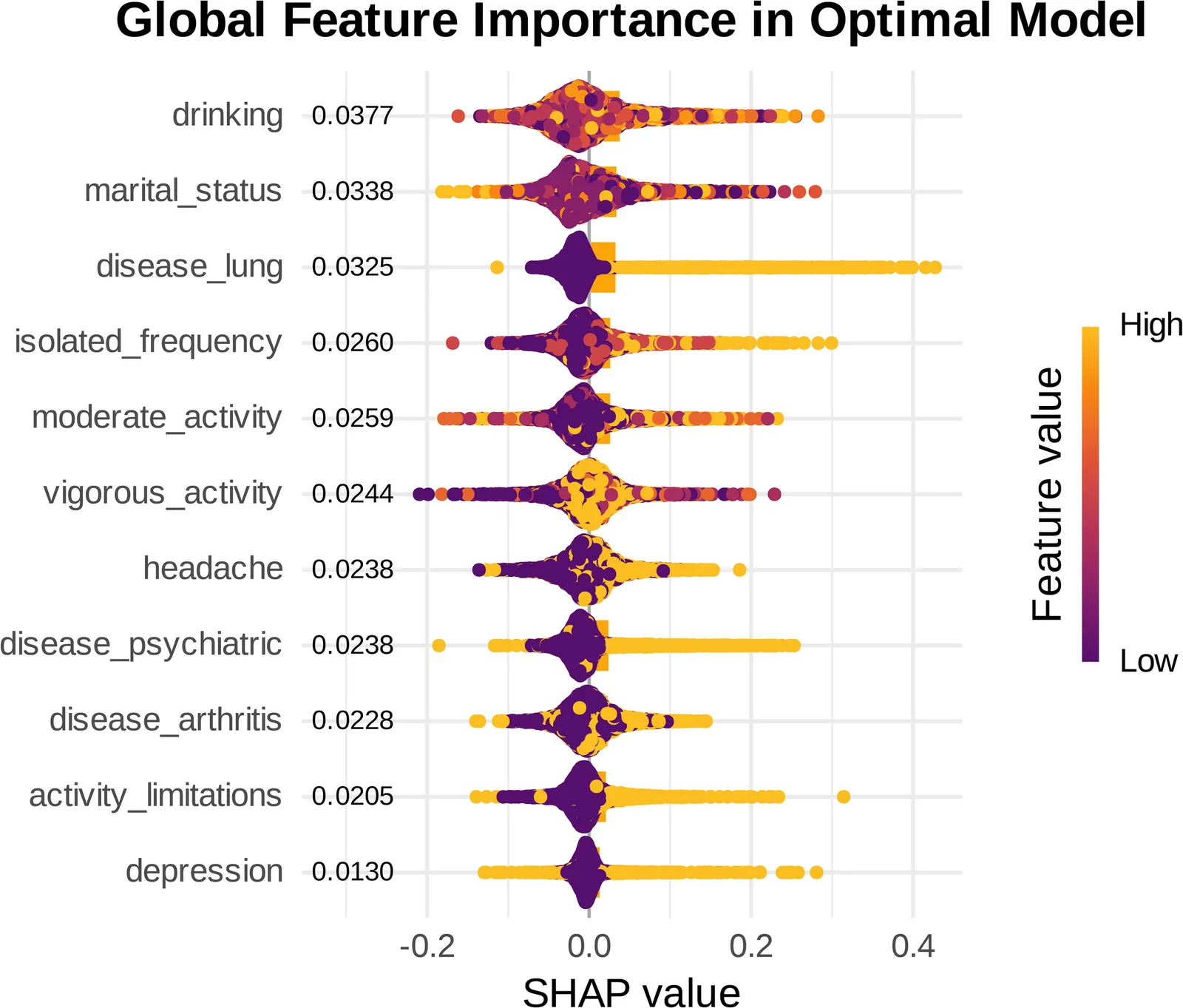
The sorting of key variables identified by the SHAP algorithm. The vertical axis of the figure represented each feature variable in the BT model. The horizontal position and color of the points indicated the SHAP value of the corresponding feature variable for each sample. The labeled values represented the importance score of each feature variable

LASSO regression analysis was performed on the 19 variables significantly associated with asthma (*p* < 0.05). At the optimal lambda value (0.00664), 14 candidate variables were retained: gender, marital status, vigorous physical activity, moderate physical activity, alcohol consumption, frequency of feeling isolated, depression, headache, activity limitations, disease lung, arthritis, cancer, stroke, and psychiatric disease (Fig. 1a-b and Additional file 2). Subsequently, eight distinct machine learning models were constructed using these 14 candidate variables to evaluate their diagnostic performance for asthma (the search range of hyperparameters for each model and the final selected values are shown in Additional file 3). Among them, the BT model demonstrated the highest micro-averaged AUC value (0.856) and macro-averaged AUC value (0.881) in the training set, indicating superior generalizability (Fig. 1c; Table 2, and Additional file 4). Based on this metrics, the BT model was selected as the optimal diagnostic model. The BT model identified 11 key variables as critical for asthma diagnosis: marital status, vigorous physical activity, moderate physical activity, alcohol consumption, frequency of feeling isolated, depression, headache, activity limitations, disease lung, arthritis, and psychiatric disease. SHAP analysis further revealed that alcohol consumption, marital status, and disease lung had the highest impact scores (> 0.03, Fig. 2).

In summary, socio-behavioral factors (marital status, alcohol use) and respiratory comorbidity (disease lung) were pivotal in asthma risk stratification. The SHAP-driven interpretability underscored the clinical relevance of these key variables in asthma screening protocols.

**Table 2:** Performance metrics of the eight machine learning prediction models (averaged over 10 runs)

Algorithm	Train AUC	Test AUC	Train LogLoss	LogLoss	Sensitivity	Specificity	Accuracy	F1	Brier	PPV	NPV	Avg Features
LR	0.65396265284112	0.635680647367915	0.405962597203762	0.41228343470991	0.994129578809211	0.0709993011879804	0.848645160401399	0.917132674541032	1.12441274954467	0.852503073572214	0.661587301587301	10.3
SGBT	0.687153522646519	0.635539206931321	0.39523683869818	0.409435685765072	0.992397088652905	0.46743535988819	0.716812860540847	0.819410316664322	0.626988832104733	0.885647460751369	0.268939573247737	8
LDA	0.645025036733282	0.63347054637828	0.633705872360316	0.636378152654142	0.762944755995705	0.0484975541579315	0.846603481691097	0.916243970240232	1.12544806607093	0.849684650436993	0.592857142857142	9.2
RF	0.833941344350877	0.620557699893715	1.04832211916567	1.49187614343319	0.992053454241737	0.130957372466806	0.822402604161091	0.900278816108646	1.18082578267576	0.855424588341947	0.326631726374761	11.2
BT	0.88132607837579	0.611024432489348	0.792902973836244	1.35723130210678	0.950242214532872	0.0762753319357093	0.84223454142396	0.913217690127671	1.14007929120102	0.852188792026056	0.473622358034123	8.8
NNET	0.697747899028014	0.602040750596317	0.389775831044873	0.461885897594236	0.983757308197112	0.0561495457721873	0.846024648712431	0.915792967049732	1.29767750747207	0.850444333066515	0.567857142857142	11.2
SVM	0.788746850464543	0.548121780522331	0.394830445564288	0.419446050028474	0.993091516525474	0.0578266946191474	0.84718572455502	0.916453891791663	1.12781413516603	0.85080826960713	0.636666666666666	10.3
DT	0.5	0.5	0.4330143 51,381,953	0.433014541595891	1	0	0.843978727470472	0.915388219068919	1.13167729372897	0.843978727470472	NA	1.2

The AUC values reported in the table are macro-averaged values, i.e., the AUC for each of the 10-fold cross validations is calculated, and then the average of the AUC values for these 10-fold cross validations is obtained

### The nomogram confirmed the predictive value of key variables and supported its application in asthma risk assessment

**Fig. 3:**
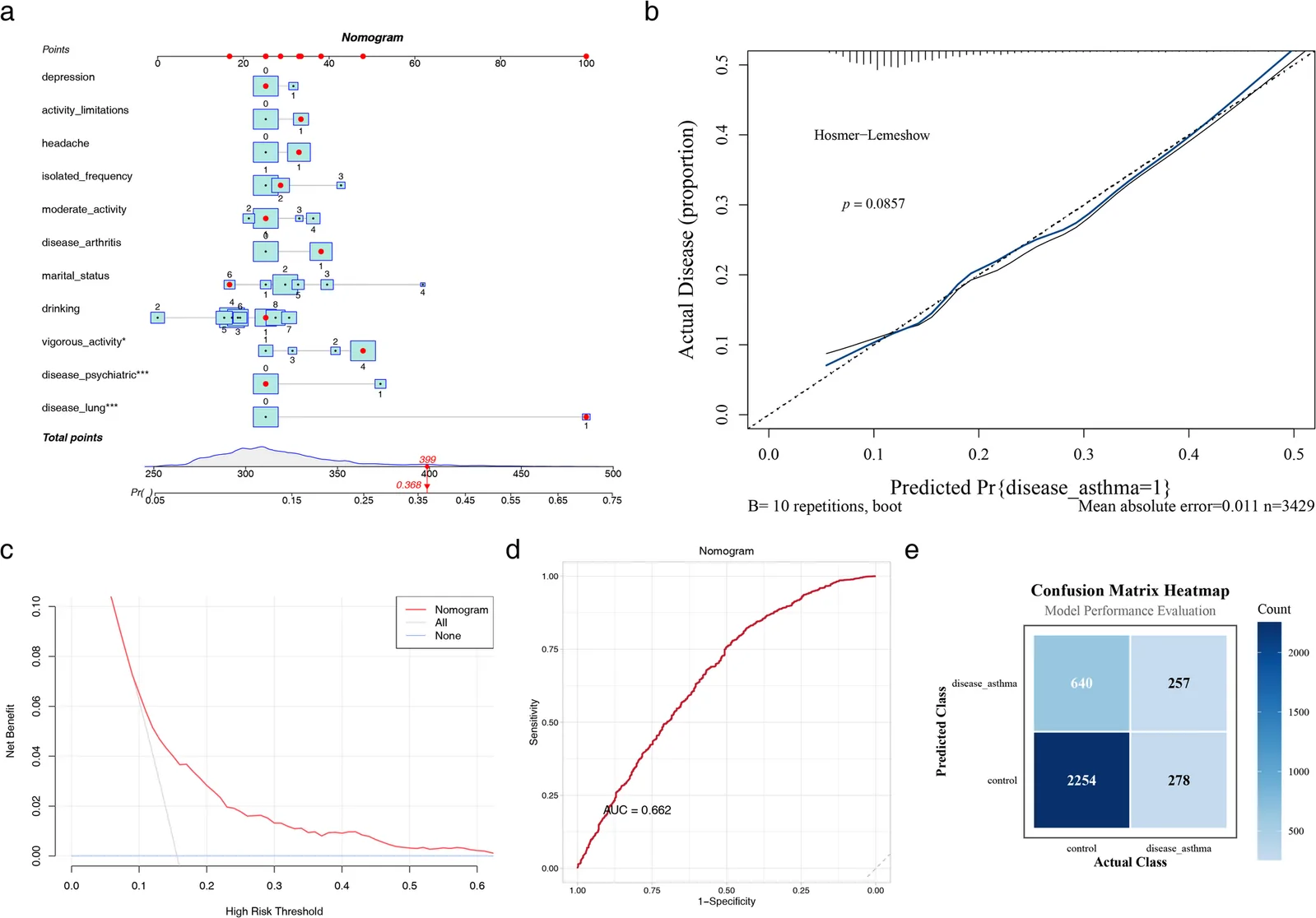
Construction and verification of the predictive model. **a** A nomogram constructed based on 11 key variables. **b** Calibration curves for nomogram. **c** The decision curve of the nomogram. **d** The ROC curve of the nomogram was plotted. The horizontal axis represented the false-positive rate (1-specificity), and the vertical axis represented the true-positive rate (sensitivity). The AUC referred to the area under the ROC curve. **e** The confusion matrix of the nomogram. The horizontal and vertical axes represented the groups, respectively. Each plot was divided into four quadrants: the top-left quadrant indicated the number of negative samples incorrectly classified as positive by the model; the bottom-left quadrant indicated the number of negative samples correctly identified as negative; the top-right quadrant indicated the number of positive samples correctly identified as positive; and the bottom-right quadrant indicated the number of positive samples incorrectly classified as negative

A nomogram was developed based on the 11 key variables identified by the BT model (Fig. 3a). Each variable was assigned a point scale proportional to its contribution to asthma risk, with longer segments indicating higher impact. The total points for an individual subject were calculated by summing the points corresponding to their specific variable values. For example, a total score of 399 corresponded to a 36.8% probability of asthma diagnosis. Notably, alcohol consumption exhibited a U-shaped association with asthma risk: moderate drinking was linked to lower risk, while minimal or heavy consumption increased risk [53]. Additionally, the calibration curve closely aligned with the reference line (slope ≈ 1), and the HL test yielded *p* = 0.0857 (Fig. 3b), indicating no significant deviation between predicted and observed probabilities. DCA demonstrated a net benefit greater than 0 across threshold probabilities, surpassing the “All” and “None” curves (Fig. 3c). The ROC curve analysis revealed an AUC of 0.662 (Fig. 3d), confirming moderate diagnostic discrimination [54]. Finally, confusion matrix analysis showed a 73.23% prediction accuracy (Fig. 3e), indicating a certain degree of predictive consistency of the nomogram. These results suggest that the nomogram constructed in this study, which integrates behavioral, psychosocial and comorbidity factors, can serve as an exploratory tool and provide a reference for improving the accuracy of clinical diagnosis in asthma risk stratification.

## Discussion

Asthma is a chronic disease characterized by airway inflammation and recurrent respiratory symptoms, with a complex etiology involving multiple physiological and environmental factors [55–58]. In recent years, alongside the accelerating aging of the population, identifying risk factors for asthma in middle-aged and older adults and establishing effective prediction models have become crucial public health priorities[59, 60]. The ELSA provides a rich data resource for such research, encompassing multidimensional information on demographics, lifestyle, psychosocial status, and health conditions, thereby supporting a comprehensive assessment of asthma-related risk factors [22, 61, 62]. Our machine learning analysis of a nationally representative cohort of older adults revealed that asthma risk is driven by a complex interplay of socio-behavioral, psychological, and comorbidity factors. Notably, alcohol consumption, marital status, and disease lung emerged as the top predictors, a finding that aligns with and extends previous research on the multifactorial nature of asthma.

The baseline analysis revealed significant differences (*p* < 0.05) between asthma and control groups across demographic characteristics, lifestyle, psychological state, and comorbidities, suggesting that asthma onset is not a singular biomedical event but rather an outcome intertwined with social, psychological, and chronic disease burdens [9, 63, 64, 13]. The higher frequencies of loneliness and depressive symptoms observed in the asthma group in this study are consistent with previous research. The underlying mechanism may involve long-term psychosocial stress disrupting the hypothalamic-pituitary-adrenal (HPA) axis and autonomic nervous system, which promotes systemic inflammatory responses, thereby exacerbating airway inflammation and hyperresponsiveness [65, 25, 66]. Similarly, insufficient physical activity, a key factor identified in this study, is not only directly associated with poor asthma control but may also exert its effects through underlying mechanisms involving regular exercise increasing anti-inflammatory cytokine levels and optimizing immune regulatory function. Recent interventional studies have further confirmed that moderate-to-vigorous aerobic exercise can effectively improve inflammatory profiles and clinical outcomes in patients with asthma [67, 11]. Furthermore, neurological comorbidities like headache/migraine are more common in individuals with asthma, possibly reflecting shared inflammatory and vascular response pathways [68, 69]. These collective differences unveil multidimensional signals for asthma risk, indicating that its determinants extend beyond traditional factors, with social and psychological health occupying central roles.

The 11 key variables and their risk hierarchies identified in this study clearly outline the multidimensional etiological map of asthma in middle-aged and elderly populations. This finding indicates that asthma risk is not driven by a single biological pathway but is shaped by the combined effects of social behavioral patterns, psychological distress, and respiratory comorbidities in the middle-aged and elderly population. A core advantage of adopting a machine learning framework lies in its ability to go beyond traditional correlation analysis and accurately quantify the relative contribution of each factor [70]. Meanwhile, machine learning models excel at capturing nonlinear relationships among variables, thereby revealing complex association patterns that are easily overlooked by conventional methods [71]. Thus, the value of this study not only lies in identifying predictor factors but also in achieving a paradigm shift from the perception of an isolated list of risks to a multisystem risk network through machine learning. This shift provides a novel theoretical basis and intervention targets for constructing precise prevention strategies integrating psychosocial support, healthy behavior promotion, and comorbidity management.

This study identified alcohol consumption, marital status, and disease lung as core functional pathways. As a direct respiratory comorbidity, disease lung can release inflammatory factors such as IL-4 and IL-5 through persistent airway inflammation and epithelial damage, activating eosinophils and mast cells to initiate or exacerbate the inflammatory cascade of asthma, serving as a direct pathological driver [72, 73]. In contrast, alcohol consumption regulates immune homeostasis through a more complex dose-dependent manner: alcoholic beverages containing sulfonamides or histamine can directly induce airway hyperresponsiveness, while its metabolite acetaldehyde can stimulate mast cells to release histamine; excessive intake inhibits T-cell function and promotes neutrophil infiltration, whereas moderate consumption may slightly suppress inflammatory signals via antioxidant pathways [74, 75]. Marital status exerts its effects indirectly primarily through psychoneuroimmunological mechanisms: marital instability or living alone may increase stress hormone levels and impair airway mucosal defense function, while stable partner relationships may form a protective effect by enhancing medication adherence and psychological resilience [63, 25]. Meanwhile, other key variables provide multidimensional risk supplements. Insufficient physical activity impairs the body’s anti-inflammatory and immune regulatory capabilities; loneliness and depression promote a systemic inflammatory state through HPA axis and autonomic nervous system dysfunction [76, 77]. Comorbidities such as headache and arthritis may collectively accumulate the risk of asthma through shared neurogenic inflammatory pathways and systemic inflammatory responses, respectively [68, 78]. In summary, the key variables identified in this study collectively form a multidimensional risk network encompassing social behaviors, psychological states, and multisystem comorbidities. This study lays an evidence-based foundation for the future development of comprehensive prevention and control strategies integrating psychosocial interventions, behavioral modifications, and comorbidity management.

In this study, a nomogram for predicting asthma risk was developed based on 11 key variables. Notably, while the nomogram demonstrated only moderate discriminatory power (AUC = 0.662), it achieved excellent calibration. In predictive modeling, discrimination (the ability to differentiate between individuals with and without the disease) and calibration (the agreement between predicted and actual risk) represent two independent metrics for evaluating model performance. A well-calibrated model can still provide relatively reliable estimates of individual risk probability, even with modest discriminatory ability. Accordingly, the nomogram established in this study remains useful as a preliminary exploratory tool for identifying individuals at high risk of asthma [79]. However, its screening performance should be further improved in future research by incorporating additional predictive factors.

The machine learning models constructed in this study, including the finally selected BT model, achieved only moderate discriminatory performance (AUC > 0.60) in the test set. This phenomenon may be attributed to several factors:

First, the data used in this study were derived from cross-sectional survey of ELSA Wave 10 data. Although the overall sample size was large, only 535 cases were in the asthma group, leading to a certain degree of class imbalance. Although stratified sampling was applied during modeling to preserve the case proportion in the training and test sets, the absolute number of the minority class (asthma group) in the test set remained relatively small. This may have resulted in insufficient sensitivity and limited the model’s ability to correctly identify positive cases.

Second, asthma is a multifactorial chronic disease whose onset and progression are influenced by a complex interplay of genetic, environmental, behavioral, psychological, and comorbid factors [80–82]. Although we included multidimensional candidate variables, some unmeasured potential confounders (e.g. air pollution exposure, occupational history, medication adherence, etc.) were not incorporated into the models, which may have constrained their predictive power.Despite these limitations in predictive performance, the key variables identified by the models—such as alcohol consumption, marital status, and chronic bronchitis—still carry important clinical and public health implications. Future studies may consider adopting longitudinal data, multi-center external validation, and more advanced deep learning or ensemble models to further improve the model’s generalizability and practical value.

This study used data from ELSA Wave 10 to systematically identify factors associated with social behavior, mental health, and multi-morbidity that may influence the development and progression of asthma, by combining LASSO selection and machine learning modeling. A nomogram for predicting the probability of asthma was further established based on the 11 key variables identified.

The findings of this study highlight the importance of viewing asthma risk in middle-aged and older adults from a multidimensional perspective, including psychosocial factors and comorbidity management. However, given the limited discriminatory performance of the prediction model, these findings should be considered exploratory.Certain limitations should also be acknowledged. Individuals with missing data were directly excluded from the analysis, which may have introduced selection bias and reduced the representativeness of the results. Although, several well-established key risk factors for asthma, such as genetic susceptibility and specific allergen exposure, were not included in the model, the absence of these strong predictors may have limited the model’s predictive accuracy (AUC). The population was restricted to individuals aged 50 years and older in the UK, whose social behavioral characteristics, healthcare environment, ethnic composition, and health background are region- and age-specific. Therefore, the findings cannot be directly generalized to other age groups, ethnicities, or regions.

To address these limitations, future studies will adopt advanced methods such as multiple imputation to handle missing data and reduce bias, provided data availability. We will also integrate multi-omics data (e.g., genomics, exposome) to incorporate genetic and environmental exposure factors and further improve predictive performance. In addition, multi-center, cross-ethnic, and cross-age external validation will be performed to evaluate the generalizability of the model. Prospective cohort studies may also be designed to further explore causal relationships among variables, providing more reliable evidence for the precise prevention and personalized intervention of asthma.

## Supplementary Information

Additional file 1.

Additional file 2.

Additional file 3.

Additional file 4.

## Data Availability

Data were obtained from the 10th wave (2021-2023) of the English Longitudinal Study of Ageing (ELSA).It’s available.

## Abbreviations

ELSA: English Longitudinal Study of Ageing
LASSO: Least Absolute Shrinkage and Selection Operator
SHAP: SHapley Additive exPlanations
BT: Bagging Tree
AUC: Area under the curve
ESRC: Economic and Social Research Council
SD: Standard deviation
IQR: Interquartile range
LR: Logistic Regression
DT: Decision Tree
SVM: Support Vector Machine
RF: Random Forest
HPA: Hypothalamic-pituitary-adrenal
LDA: Linear Discriminant Analysis
NNET: Neural Network
SGBT: Stochastic Gradient Boosting Tree
PPV/NPV: Positive/negative predictive value
ROC: Receiver operating characteristic

## References

[1] Wang R, Murray CS, Fowler SJ, Simpson A, Durrington HJ. Asthma diagnosis: into the fourth dimension. Thorax. 2021;76(6):624–31. 33504564 10.1136/thoraxjnl-2020-216421 PMC8223645

[2] Varricchi G, Ferri S, Pepys J, Poto R, Spadaro G, Nappi E, et al. Biologics and airway remodeling in severe asthma. Allergy. 2022;77(12):3538–52. 35950646 10.1111/all.15473 PMC10087445

[3] Bousquet J, Schünemann HJ, Sousa-Pinto B, Zuberbier T, Togias A, Samolinski B, et al. Concepts for the Development of Person-Centered, Digitally Enabled, Artificial Intelligence-Assisted ARIA Care Pathways (ARIA 2024). The journal of allergy and clinical immunology. Pract. 2024;12(10):2648–e26682642. 10.1016/j.jaip.2024.06.040 38971567

[4] Fan Z, Xu M, Chen S, Wang J, Gong Y, Feng X, et al. Association of Socioeconomic Status and a Broad Combination of Lifestyle Factors With Adult-Onset Asthma: A Cohort Study. The journal of allergy and clinical immunology. Pract. 2024;12(8):2066–73. 10.1016/j.jaip.2024.04.009 38631523

[5] Renz H, Skevaki C. Early life microbial exposures and allergy risks: opportunities for prevention. Nat Rev Immunol. 2021;21(3):177–91. 32918062 10.1038/s41577-020-00420-y

[6] Melén E, Zar HJ, Siroux V, Shaw D, Saglani S, Koppelman GH, et al. Asthma Inception: Epidemiologic Risk Factors and Natural History Across the Life Course. Am J Respir Crit Care Med. 2024;210(6):737–54. 38981012 10.1164/rccm.202312-2249SO PMC11418887

[7] Thio CL, Chang YJ. The modulation of pulmonary group 2 innate lymphoid cell function in asthma: from inflammatory mediators to environmental and metabolic factors. Exp Mol Med. 2023;55(9):1872–84. 37696890 10.1038/s12276-023-01021-0 PMC10545775

[8] Li H, Tang X, Guo X, Zhang M, Zhang M, Nie J, et al. Association of dietary patterns with chronic respiratory health among U.S. adults. Front Immunol. 2024;15:1457860. 39712005 10.3389/fimmu.2024.1457860 PMC11659122

[9] Chowdhury NU, Guntur VP, Newcomb DC, Wechsler ME. Sex and gender in asthma. European respiratory review: an official Journal of the European Respiratory Society. 2021;30(162). 10.1183/16000617.0067-2021 PMC8783601 34789462

[10] Kim FS, Rocha JL, Lunardi AC, Bos DSG, Santos EA, Marques da Silva CCB, et al. Effects of Combined Aerobic and Breathing Exercises on Asthma Control: A Randomized Controlled Trial. The journal of allergy and clinical immunology. Pract. 2024;12(12):3328–36. 10.1016/j.jaip.2024.07.036 39216805

[11] McLoughlin RF, Clark VL, Urroz PD, Gibson PG, McDonald VM. Increasing physical activity in severe asthma: a systematic review and meta-analysis. Eur Respir J. 2022;60(6). 10.1183/13993003.00546-2022 PMC9753478 35896208

[12] Kuder MM, Clark M, Cooley C, Prieto-Centurion V, Danley A, Riley I, et al. A Systematic Review of the Effect of Physical Activity on Asthma Outcomes. The journal of allergy and clinical immunology. Pract. 2021;9(9):3407–e34213408. 10.1016/j.jaip.2021.04.048 PMC8434961 33964510

[13] Ledford DK, Kim TB, Ortega VE, Cardet JC. Asthma and respiratory comorbidities. J Allergy Clin Immunol. 2025;155(2):316–26. 39542142 10.1016/j.jaci.2024.11.006 PMC12180284

[14] Gibson PG, McDonald VM, Granchelli A, Olin JT. Asthma and Comorbid Conditions-Pulmonary Comorbidity. The journal of allergy and clinical immunology. Pract. 2021;9(11):3868–75. 10.1016/j.jaip.2021.08.028 34492401

[15] Dahl R. Systemic side effects of inhaled corticosteroids in patients with asthma. Respir Med. 2006;100(8):1307–17. 16412623 10.1016/j.rmed.2005.11.020

[16] Rolla G. Why Current Therapy Does Not Cure Asthma. Is It Time to Move Towards a One Health Approach? J asthma allergy. 2023;16:933–6. 37692125 10.2147/JAA.S429646 PMC10488599

[17] Barnes PJ. Corticosteroid resistance in patients with asthma and chronic obstructive pulmonary disease. J Allergy Clin Immunol. 2013;131(3):636–45. 23360759 10.1016/j.jaci.2012.12.1564

[18] Chen S, Kuper H. Tracing the temporal trends of modifiable risk factors in dementia: insights from the English Longitudinal Study of Ageing (2004–2019). Lancet (London England). 2023;402(Suppl 1):S34. 37997075 10.1016/S0140-6736(23)02078-0

[19] Hackett RA, Hudson JL, Chilcot J. Loneliness and type 2 diabetes incidence: findings from the English Longitudinal Study of Ageing. Diabetologia. 2020;63(11):2329–38. 32929525 10.1007/s00125-020-05258-6 PMC7527325

[20] Beard JR, Hanewald K, Si Y, Amuthavalli Thiyagarajan J, Moreno-Agostino D. Cohort trends in intrinsic capacity in England and China. Nat aging. 2025;5(1):87–98. 39702725 10.1038/s43587-024-00741-w PMC11754101

[21] Xiong Y, Zhong Q, Zhang Y, Liu Z, Wang X. The association between circadian syndrome and chronic kidney disease in an aging population: a 4-year follow-up study. Front Endocrinol. 2024;15:1338110. 10.3389/fendo.2024.1338110 PMC11082579 38737554

[22] Ji X, Zhu Y, Ahmadizar F, Gao H, Sun D, Zhang J et al. Cognitive decline before and after incident chronic respiratory disease. Geroscience. 2025. 10.1007/s11357-025-01754-y PMC12638625 40540151

[23] Sarwar MR, McDonald VM, Abramson MJ, Paul E, George J. Treatable traits in an English cohort: prevalence and predictors of future decline in lung function and quality of life in COPD. ERJ Open Res. 2021;7(2). 10.1183/23120541.00934-2020 PMC8165376 34084787

[24] Li P, Zhang C, Gao S, Zhang Y, Liang X, Wang C, et al. Association Between Daily Internet Use and Incidence of Chronic Diseases Among Older Adults: Prospective Cohort Study. J Med Internet Res. 2023;25:e46298. 37459155 10.2196/46298 PMC10390981

[25] Leukel PJ, Piette JD, Lee AA. Impact of Loneliness and Social Support on Acute Health Service Use and Symptom Exacerbation Among Adults with Asthma and COPD. J Clin Psychol Med Settings. 2025;32(2):375–84. 39369147 10.1007/s10880-024-10046-0 PMC12081591

[26] McKernan KE, Pilier EH, Newcomb DC. Sex Differences in Asthma Pathogenesis. Immunol Rev. 2026;337(1). 10.1111/imr.70100 PMC12783869 41508394

[27] Miligkos M, Oh J, Kwon R, Konstantinou GΝ, Kim S, Yon DK, et al. Epidemiology of asthma across the ages. Ann Allergy Asthma Immunol. 2025;134(4):376–e384313. 39674277 10.1016/j.anai.2024.12.004

[28] Kamga A, Rochefort-Morel C, Guen YL, Ouksel H, Pipet A, Leroyer C. Asthma and smoking: A review. Respiratory Med Res. 2022;82:100916. 10.1016/j.resmer.2022.100916 35901579

[29] Price OJ, Simpson AJ. Exercise and asthma – trigger or treatment? Respir Med. 2023;213:107247. 37086818 10.1016/j.rmed.2023.107247

[30] Nielsen LB, Johansen MO, Riddersholm SJ, Weinreich UM. The association between alcohol consumption and pulmonary function: a scoping review. Eur Respiratory Rev. 2024;33(172):230233. 10.1183/16000617.0233-2023 PMC11078152 38719738

[31] Smith KJ, Gavey S, Riddell NE, Kontari P, Victor C. The association between loneliness, social isolation and inflammation: A systematic review and meta-analysis. Neurosci Biobehavioral Reviews. 2020;112:519–41. 10.1016/j.neubiorev.2020.02.002 32092313

[32] Van Bogart K, Engeland CG, Sliwinski MJ, Harrington KD, Knight EL, Zhaoyang R et al. The Association Between Loneliness and Inflammation: Findings From an Older Adult Sample. Front Behav Neurosci. 2022;15. 10.3389/fnbeh.2021.801746 PMC8787084 35087386

[33] Palumbo ML, Prochnik A, Wald MR, Genaro AM. Chronic Stress and Glucocorticoid Receptor Resistance in Asthma. Clin Ther. 2020;42(6):993–1006. 32224031 10.1016/j.clinthera.2020.03.002

[34] Liu X, Plana-Ripoll O, McGrath JJ, Petersen LV, Dharmage SC, Momen NC. Bidirectional Associations Between Asthma and Types of Mental Disorders. J Allergy Clin Immunology: Pract. 2023;11(3):799–e808714. 36481421 10.1016/j.jaip.2022.11.027

[35] Panos A, Mavridis D. TableOne: an online web application and R package for summarising and visualising data. Evid Based Ment Health. 2020;23(3):127–30. 32665250 10.1136/ebmental-2020-300162 PMC10231609

[36] Friedman J, Hastie T, Tibshirani R. Regularization Paths for Generalized Linear Models via Coordinate Descent. J Stat Softw. 2010;33(1):1–22. 20808728 PMC2929880

[37] Goncharova IA, Nazarenko MS, Babushkina NP, Markov AV, Pecherina TB, Kashtalap VV, et al. [Genetic Predisposition to Early Myocardial Infarction]. Mol Biol. 2020;54(2):224–32. 10.31857/S0026898420020044 32392191

[38] Andersson B, Langen B, Liu P, Dávila López M. Development of a machine learning framework for radiation biomarker discovery and absorbed dose prediction. Front Oncol. 2023;13:1156009. 37256187 10.3389/fonc.2023.1156009 PMC10225714

[39] Shi H, Yuan X, Liu G, Fan W. Identifying and Validating GSTM5 as an Immunogenic Gene in Diabetic Foot Ulcer Using Bioinformatics and Machine Learning. J Inflamm Res. 2023;16:6241–56. 38145013 10.2147/JIR.S442388 PMC10748866

[40] Li S, Que Y, Yang R, He P, Xu S, Hu Y. Construction of Osteosarcoma Diagnosis Model by Random Forest and Artificial Neural Network. J personalized Med. 2023;13(3). 10.3390/jpm13030447 PMC10056981 36983630

[41] Sharma VK, Glick J, Liao Q, Shen C, Vouros P. GenoMass software: a tool based on electrospray ionization tandem mass spectrometry for characterization and sequencing of oligonucleotide adducts. J Mass Spectrom. 2012;47(4):490–501. 22689626 10.1002/jms.2054 PMC3375619

[42] Beck MW. NeuralNetTools: Visualization and Analysis Tools for Neural Networks. J Stat Softw. 2018;85(11):1–20. 30505247 10.18637/jss.v085.i11 PMC6262849

[43] Salditt M, Humberg S, Nestler S. Gradient Tree Boosting for Hierarchical Data. Multivar Behav Res. 2023;58(5):911–37. 10.1080/00273171.2022.2146638 36602080

[44] Robin X, Turck N, Hainard A, Tiberti N, Lisacek F, Sanchez JC, et al. pROC: an open-source package for R and S + to analyze and compare ROC curves. BMC Bioinformatics. 2011;12:77. 21414208 10.1186/1471-2105-12-77 PMC3068975

[45] Huang T, Yang R, Shen L, Feng A, Li L, He N et al. Deep transfer learning to quantify pleural effusion severity in chest X-rays. BMC Med Imaging. 2022;22(1). 10.1186/s12880-022-00827-0 PMC9137166 35624426

[46] Lin N, Shi Y, Ye M, Zhang Y, Jia X. Deep transfer learning radiomics for distinguishing sinonasal malignancies: a preliminary MRI study. Future Oncol. 2025;21(8):975–82. 39991909 10.1080/14796694.2025.2469486 PMC11938957

[47] Guo C, Yang X, Li L. Pyroptosis-Related Gene Signature Predicts Prognosis and Response to Immunotherapy and Medication in Pediatric and Young Adult Osteosarcoma Patients. J Inflamm Res. 2024;17:417–45. 38269108 10.2147/JIR.S440425 PMC10807455

[48] Lin G, Gao Z, Wu S, Zheng J, Guo X, Zheng X, et al. scRNA-seq revealed high stemness epithelial malignant cell clusters and prognostic models of lung adenocarcinoma. Sci Rep. 2024;14(1):3709. 38355636 10.1038/s41598-024-54135-4 PMC10867035

[49] Zhu H, Hu H, Hao B, Zhan W, Yan T, Zhang J, et al. Insights into a Machine Learning-Based Palmitoylation-Related Gene Model for Predicting the Prognosis and Treatment Response of Breast Cancer Patients. Technol Cancer Res Treat. 2024;23:15330338241263434. 39205467 10.1177/15330338241263434 PMC11363247

[50] Liu C, He Y, Luo J. Application of Chest CT Imaging Feature Model in Distinguishing Squamous Cell Carcinoma and Adenocarcinoma of the Lung. Cancer Manage Res. 2024;16:547–57. 10.2147/CMAR.S462951 PMC11162187 38855330

[51] Chen Y, Ju H, Xie K, Zhao X. Association of inflammatory score with all-cause and cardiovascular mortality in patients with metabolic syndrome: NHANES longitudinal cohort study. Front Immunol. 2024;15:1410871. 39011047 10.3389/fimmu.2024.1410871 PMC11246876

[52] Mangiola S, Doyle MA, Papenfuss AT. Interfacing Seurat with the R tidy universe. Bioinf (Oxford England). 2021;37(22):4100–7. 10.1093/bioinformatics/btab404 PMC9502154 34028547

[53] Lieberoth S, Backer V, Kyvik KO, Skadhauge LR, Tolstrup JS, Grønbæk M, et al. Intake of alcohol and risk of adult-onset asthma. Respir Med. 2012;106(2):184–8. 22129491 10.1016/j.rmed.2011.11.004

[54] Silva AM, Levy J, De Carli E, Cacau LT, de Alvarenga JFR, Benseñor IJM, et al. Biomarker panels for fruit intake assessment: a metabolomics analysis in the ELSA-Brasil study. Metabolomics: Official J Metabolomic Soc. 2024;20(4):88. 10.1007/s11306-024-02145-8 39073486

[55] Olsthoorn SEM, van Krimpen A, Hendriks RW, Stadhouders R. Chronic Inflammation in Asthma: Looking Beyond the Th2 Cell. Immunol Rev. 2025;330(1):e70010. 40016948 10.1111/imr.70010 PMC11868696

[56] Nguyen H, Nasir M. Management of Chronic Asthma in Adults. Med Clin N Am. 2024;108(4):629–40. 38816107 10.1016/j.mcna.2023.08.007

[57] Xu Q, Zhou Q, Chen J, Li T, Ma J, Du R, et al. The incidence of asthma attributable to temperature variability: An ecological study based on 1990–2019 GBD data. Sci Total Environ. 2023;904:166726. 37659541 10.1016/j.scitotenv.2023.166726

[58] Miller RL, Grayson MH, Strothman K. Advances in asthma: New understandings of asthma’s natural history, risk factors, underlying mechanisms, and clinical management. J Allergy Clin Immunol. 2021;148(6):1430–41. 34655640 10.1016/j.jaci.2021.10.001

[59] Ford ML, Ruwanpathirana A, Lewis BW, Britt RD. Jr. Aging-Related Mechanisms Contribute to Corticosteroid Insensitivity in Elderly Asthma. Int J Mol Sci. 2023;24(7). 10.3390/ijms24076347 PMC10093993 37047327

[60] Polivka BJ, Huntington-Moskos L, Antimisiaris DE, Cavallazzi RS, Folz RJ. Phenotyping older adults with asthma by means of cluster analysis. Ann Allergy Asthma Immunol. 2022;129(3):376–8. 35697194 10.1016/j.anai.2022.06.003 PMC10859917

[61] Ji T, Lv Y, Yang J, Diao X, Gu J. Association between depression and asthma: insight from observational and genetic evidence. BMC Psychiatry. 2025;25(1):786. 40796827 10.1186/s12888-025-07245-w PMC12341131

[62] Yu R, Chen Z, Zou L. The impact of sleep quality on asthma incidence in middle-aged and older adults: evidence from a prospective cohort study. Front public health. 2025;13:1646053. 40823227 10.3389/fpubh.2025.1646053 PMC12352347

[63] Vennu V, Abdulrahman TA, Alenazi AM, Bindawas SM. Associations between social determinants and the presence of chronic diseases: data from the osteoarthritis Initiative. BMC Public Health. 2020;20(1):1323. 32867751 10.1186/s12889-020-09451-5 PMC7461338

[64] Born CDC, Bhadra R, D’Souza G, Kremers SPJ, Sambashivaiah S, Schols A et al. Combined Lifestyle Interventions in the Prevention and Management of Asthma and COPD: A Systematic Review. Nutrients. 2024;16(10). 10.3390/nu16101515 PMC11124109 38794757

[65] Meng L, Xu R, Li J, Hu J, Xu H, Wu D, et al. The silent epidemic: exploring the link between loneliness and chronic diseases in China’s elderly. BMC Geriatr. 2024;24(1):710. 39187783 10.1186/s12877-024-05163-2 PMC11346041

[66] Sipowicz K, Podlecka M, Mokros Ł, Pietras T, Łuczyńska K. The feeling of loneliness and the sense of meaning in life in patients with various levels of bronchial asthma control. J asthma: official J Association Care Asthma. 2023;60(7):1402–8. 10.1080/02770903.2022.2151465 36440842

[67] Ang J, Moussa R, Shaikh S, Mele S. Effects of aerobic exercise on asthma control and quality of life in adults: a systematic review. J asthma: official J Association Care Asthma. 2023;60(5):845–55. 10.1080/02770903.2022.2103429 35862617

[68] Chin HL, Cheong KK. Association between asthma and headache: Findings from the NHANES 2001–2004. Clin Respir J. 2023;17(8):799–804. 10.1111/crj.13664 PMC10435949 37431155

[69] Kang LL, Chen PE, Tung TH, Chien CW. Association Between Asthma and Migraine: A Systematic Review and Meta-Analysis of Observational Studies. Front allergy. 2021;2:741135. 35386963 10.3389/falgy.2021.741135 PMC8974722

[70] Rajula HSR, Verlato G, Manchia M, Antonucci N, Fanos V. Comparison of Conventional Statistical Methods with Machine Learning in Medicine: Diagnosis, Drug Development, and Treatment. Med (Kaunas). 2020;56(9). 10.3390/medicina56090455 PMC7560135 32911665

[71] Julkunen H, Rousu J. Comprehensive interaction modeling with machine learning improves prediction of disease risk in the UK Biobank. Nat Commun. 2025;16(1):6620. 40681488 10.1038/s41467-025-61891-y PMC12274440

[72] Alsayed AR, Abu-Samak MS, Alkhatib M. Asthma-COPD Overlap in Clinical Practice (ACO_CP 2023): Toward Precision Medicine. J personalized Med. 2023;13(4). 10.3390/jpm13040677 PMC10146260 37109063

[73] Zhu M, Chen A. Epidemiological characteristics of asthma-COPD overlap, its association with all-cause mortality, and the mediating role of depressive symptoms: evidence from NHANES 2005–2018. BMC Public Health. 2024;24(1):1423. 38807148 10.1186/s12889-024-18911-1 PMC11134654

[74] Wüthrich B. Allergic and intolerance reactions to wine. Allergologie select. 2018;2(1):80–8. 31826033 10.5414/ALX01420E PMC6883207

[75] Kawano T, Matsuse H, Kondo Y, Machida I, Saeki S, Tomari S, et al. Acetaldehyde induces histamine release from human airway mast cells to cause bronchoconstriction. Int Arch Allergy Immunol. 2004;134(3):233–9. 15178893 10.1159/000078771

[76] Caulfield JI. Anxiety, depression, and asthma: New perspectives and approaches for psychoneuroimmunology research. Brain, behavior, & immunity - health. 2021;18:100360. 10.1016/j.bbih.2021.100360 PMC8502834 34661176

[77] Brown ES. The Complex Relationship Between Asthma and Psychiatric Illnesses. The journal of allergy and clinical immunology. Pract. 2023;11(3):809–10. 10.1016/j.jaip.2023.01.015 36894279

[78] Kim JG, Kang J, Lee JH, Koo HK. Association of rheumatoid arthritis with bronchial asthma and asthma-related comorbidities: A population-based national surveillance study. Front Med. 2023;10:1006290. 10.3389/fmed.2023.1006290 PMC10036351 36968830

[79] Alba AC, Agoritsas T, Walsh M, Hanna S, Iorio A, Devereaux PJ, et al. Discrimination and Calibration of Clinical Prediction Models. JAMA. 2017;318(14):1377. 29049590 10.1001/jama.2017.12126

[80] Stubbs MA, Clark VL, Gibson PG, Yorke J, McDonald VM. Associations of symptoms of anxiety and depression with health-status, asthma control, dyspnoea, dysfunction breathing and obesity in people with severe asthma. Respir Res. 2022;23(1). 10.1186/s12931-022-02266-5 PMC9743554 36510255

[81] Johansson H, Mersha TB, Brandt EB, Khurana Hershey GK. Interactions between environmental pollutants and genetic susceptibility in asthma risk. Curr Opin Immunol. 2019;60:156–62. 31470287 10.1016/j.coi.2019.07.010 PMC6800636

[82] Hizawa N. The understanding of asthma pathogenesis in the era of precision medicine. Allergology Int. 2023;72(1):3–10. 10.1016/j.alit.2022.09.001 36195530

