# Supplementary material for: Identifying predictor factors for asthma using machine learning: evidence from the English Longitudinal Study of Ageing
Source: BMC Public Health. 2026 Jul 3;26:2652. doi: 10.1186/s12889-026-28253-9 (PMC13599194; doi:10.1186/s12889-026-28253-9)
Supplement: Supplementary file 1 [file 12889_2026_28253_MOESM3_ESM.docx]

**Additional file 3** Plotting the search range of hyperparameters and the final selected values of 8 machine learning models.
